# Carbon nanotubes/lithium ferrite nanocomposites: magnetic and electrochemical optimization for enhanced H_2_O_2_ sensing

**DOI:** 10.1039/d5ra04502a

**Published:** 2025-09-15

**Authors:** Emtinan Ouda, Nehad Yousf, Amir Elzwawy, Hend S. Magar, Rabeay Y. A. Hassan, Magdy El-Ashry, El-Shazly M. Duraia

**Affiliations:** a Physics Department, Faculty of Science, Suez Canal University Ismailia 41522 Egypt; b Ceramics Department, Advanced Materials Technology and Mineral Resources Research Institute, National Research Centre (NRC) 33 El Bohouth St., Dokki Giza 12622 Egypt aa.elzwawy@nrc.sci.eg elzwawy1@gmail.com; c Applied Organic Chemistry Department, National Research Centre (NRC) 33 El Bohouth St., Dokki Giza 12622 Egypt; d Biosensors Research Lab, Zewail City of Science and Technology 6th October City Giza 12578 Egypt; e Laboratories Manager, Faculty of Medicine, Suez Canal University Ismailia 41522 Egypt

## Abstract

Hydrogen peroxide (H_2_O_2_) is a ubiquitous molecule in biological systems, but at elevated concentrations, it exhibits cytotoxicity, necessitating precise monitoring for both biomedical and analytical applications. In this work, we report a cost-effective strategy for synthesizing carbon nanotube/lithium ferrite (CNTs/LFO) nanocomposites with different LFO doping levels (0.5%, 1%, and 2%) for non-enzymatic H_2_O_2_ sensing. The nanocomposites were fabricated *via* a citrate–gel auto-combustion route, yielding uniformly dispersed structures. X-ray diffraction (XRD) and field emission scanning electron microscopy (FE-SEM) confirmed the presence of a crystalline ferrite phase with nanoplate particles averaging ∼50 nm. Vibrating sample magnetometry (VSM) revealed a maximum saturation magnetization of 25 emu g^−1^ for the 2% LFO composition. Electrochemical characterization using cyclic voltammetry (CV) demonstrated superior H_2_O_2_ sensing activity of CNTs/LFO compared to pure LFO, attributed to accelerated electron transfer at the CNTs-modified interface. The optimized electrode exhibited excellent stability, a low detection limit of 0.005 μM, and a wide linear response range of 0.1–500 μM. These results highlight CNTs/LFO nanocomposites as highly promising candidates for advanced H_2_O_2_ sensing and related electrochemical applications.

## Abstract 2

Hydrogen peroxide (H_2_O_2_) is a ubiquitous molecule in biological systems, but at elevated concentrations, it exhibits cytotoxicity, necessitating precise monitoring for both biomedical and analytical applications.

## Graphical abstract

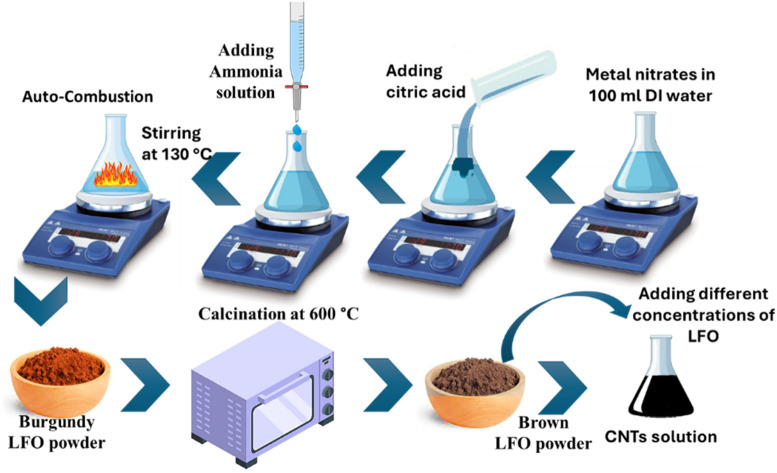

## 1. Introduction

Hydrogen peroxide (H_2_O_2_) plays a crucial role across various industries, including food production, pharmaceutical preparations, and medical treatments, nominating it as one of the most significant industrial chemicals.^1–3^ Consequently, there is a pressing need for the development of rapid and straightforward sensors for H_2_O_2_ detection.^4–6^ Among the various techniques explored for this purpose, electrochemical sensors^7–11^ have attracted a lot of attention because of their intrinsic advantages (*e.g.* high sensitivity,^12^ selectivity,^13^ low cost, and ease of use).^14–19^ Traditionally, noble metal nanoparticles, such as gold, silver, palladium, and platinum, have been employed to modify electrodes for efficient H_2_O_2_ detection.^20–23^

However, the high cost, limited availability, and poor selectivity of these materials have restricted their broader applicability in H_2_O_2_ sensing.^24–26^ Thus, there is a pressing necessity to provide new materials that can enhance H_2_O_2_ detection capabilities while overcoming these limitations. Li *et al.* (2025), have strategies for enhancing H_2_O_2_ production through semiconductor composite design, implying the connection between their S-scheme W_18_O_49_/ZnIn_2_S_4_ photocatalyst.^27^ The work by Du *et al.* (2025) on H_2_O_2_-assisted photocatalytic pollutant removal using Bi-based composites provides valuable insight into the interaction of catalytic nanocomposites with H_2_O_2_.^28^

Lithium ferrite (LFO) has recently gained significant attention due to its diverse applications, including its use as a semiconductor photocatalyst,^29^ sensor material,^30^ cancer treatment agent,^31^ magnetic-optical component,^32^ and antibacterial agent.^33^ Despite its versatility, the electrochemical performance of LFO is often compromised by spontaneous agglomeration and low electrical conductivity, which limits its practical applications in sensors. Therefore, enhancing its conductivity is critical for improving its electrochemical performance.

To address this challenge, carbon nanotubes (CNTs) have appeared as a promising solution owing to their significant nanostructure and exceptional electrical properties.^24,34,35^ The conjugation between CNTs and metal nanoparticles is beneficial in reducing agglomeration^36,37^ and accelerating the transfer of ions and electrons. The characterization, preparation, and potential uses of spinel ferrite materials for several technological fields have been covered in numerous studies. For example, Thanasak Sathiwitayakul,^38^ Josué M. Gonçalvesand Ganjali,^39^ and Kaidi Wu^40^ reviewed the sensing performances of spinel ferrite-based electrochemical sensors.

Ma *et al.* (2025) investigated the MWCNTs–Zn_0_._3_Cd_0_·_7_S/Pd composites for photocatalytic H_2_ evolution, providing valuable insights into the role of CNTs-based hybrid structures in enhancing electrochemical and catalytic performance.^41^

Conversely, a few studies have concentrated on the functionalization of lithium ferrite-based CNTs for constructing gas sensors. Ranga *et al.*^42^ summarized the outcomes on carbon material/ferrite-based core–shell structures for gas sensor applications.

S. Sahoo *et al.* reported a non-enzymatic hydrogen peroxide sensor based on CoFe_2_O_4_/CNTs nanocomposites. However, to the best of our knowledge, no studies have yet reported the highly promising performance of CNTs/LFO nanocomposites as electroactive layers in electrochemical sensors.^43^

In this study, we report the preparation and characterization of carbon nanotubes (CNTs)-doped lithium ferrite (LFO) nanocomposites to investigate their magnetic and electrochemical properties. The integration of CNTs with LFO is intended to enhance electrical conductivity and evaluate their potential application in efficient non-enzymatic hydrogen peroxide (H_2_O_2_) sensing. This work provides a foundation for the development of advanced sensor materials with potential applications in diverse fields, particularly in the monitoring of H_2_O_2_ in industrial and biomedical contexts.

## 2. Experimental section

### 2.1 Materials and characterization

Powder of carbon nanotubes (CNTs) was procured from Nanoridge, Houston, USA. Ferric nitrate (Fe(NO_3_)_3_·9H_2_O) and lithium nitrate (LiNO_3_·3H_2_O) were sourced from Loba-Chemie Pvt-Ltd, India. Additional chemicals, such as (K_4_Fe(CN)_6_) potassium ferrocyanide, (K_3_[Fe(CN)_6_]) potassium ferricyanide, (KCl) potassium chloride, and hydrogen peroxide (H_2_O_2_), were obtained from Sigma-Aldrich. Phosphate-buffered saline (PBS, tablets with a pH of 7.4) was purchased from MPBio, USA.

X-ray diffraction (XRD) analysis was carried out using a Bruker D8 diffractometer with CuKα radiation having a wavelength of 1.5481 Å at room temperature. The apparent morphology of the produced nanocomposites was investigated using scanning electron microscopy (SEM, Philips XL 30) at an accelerating voltage of 30 kV and magnification of 10× up to 400.000×. The magnetic characteristics of the as-synthesised samples were investigated using a vibrating sample magnetometer (VSM; LakeShore −7410-USA). At ambient room temperature, all VSM measurements were performed. Screen-printed electrodes (SPEs), which encompass an apparatus with three electrodes that include the counter, working, and reference electrodes, were utilized in electrochemical investigations. A PalmSens 4 potentiostat electrochemical workstation was used to perform the electrochemical measurements.

### 2.2 Lithium ferrite (LFO) nanoparticle synthesis

**Fig. 1:**
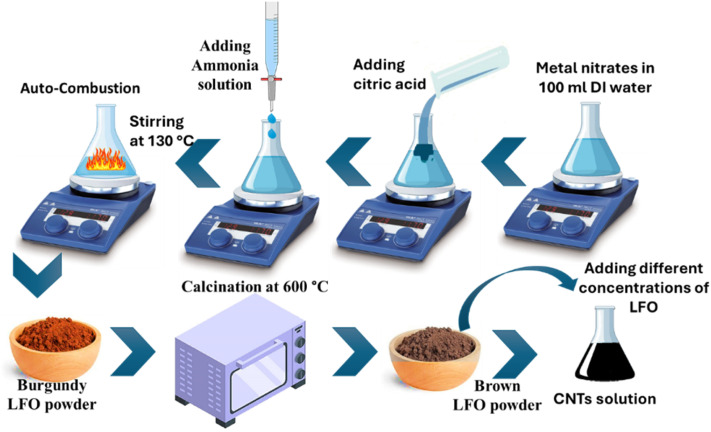
Schematic of the preparation procedure for carbon nanomaterial-based lithium ferrite (LFO) nanocomposites. The figure outlines the key steps involved in synthesizing the nanocomposite materials, including the initial mixing of precursors, followed by calcination and reduction processes to form the final carbon nanotubes/lithium ferrite nanocomposites.

Lithium ferrite (LiFe_5_O_8_; LFO) nanoparticles were chemically synthesized using the reported protocol (a simple citrate-gel auto-combustion approach^44^), as depicted in Fig. 1. In a typical procedure, ferric nitrate (Fe(NO_3_)_3_·9H_2_O) and lithium nitrate (LiNO_3_·3H_2_O) were disintegrated in 100 mL of deionized (DI) water and stirred for 15 minutes. After dissolution, citric acid was introduced as a chelating agent at a 1 : 1 molar ratio with respect to the metal ions. The pH of the solution was tuned to 7.0 using drops of ammonia solution (33%). The solution was continuously stirred and heated at 130 °C until it transformed into a xerogel.

The xerogel was then subjected to combustion inside an oven, which triggered the formation of ferrite nanostructures. This sol–gel route encourages an anionic redox reaction in the xerogel through an exothermic, self-sustaining response. The combustion process sources a quick gas emission together with a notable mass loss, resulting in the formation of ferrite nano-powder. To further enhance the properties of lithium ferrite (LFO), the powder underwent additional heat treatment. Specifically, the LFO powder, which appeared burgundy in color, was placed in a furnace and sintered in a crucible for four hours at 600 °C, turning the final product brown.

### 2.3 Carbon nanomaterial-based lithium ferrite (CNTs/LFO) synthesis

The reaction was conducted using a microwave at high power for 20 minutes, a fast and precise method for facilitating the reaction between carbon nanotubes (CNTs) and lithium ferrite (LFO). A suspension of carbon nanotubes was prepared at a concentration of one gram per milliliter, which was stirred with a magnetic stirrer. Varying amounts of LFO were then added to this CNTs dispersion to create a series of nanocomposites with different ferrite concentrations under sustained stirring to ensure homogeneity. The LFO concentrations are 0.5, 1.0, and 2.0 mg mL^−1^, and the samples are denoted as CNTs/LFO (0.5%), CNTs/LFO (1%) and CNTs/LFO (2%), respectively.

### 2.4 Modification of printed electrodes with CNTs/LFO

**Fig. 2:**
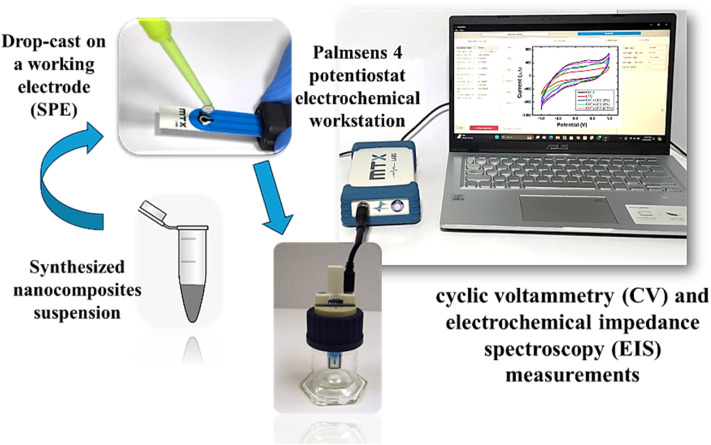
Simple modification of screen-printed electrodes (SPEs) with the synthesized nanomaterials for electrochemical characterization and sensing applications.

Screen-printed electrodes (SPEs) were modified with the synthesized nanocomposites as follows: 10 mg mL^−1^ of CNTs/LFO nanocomposite was dispersed in 1.0 mL of double-distilled water and ultrasonicated for 30 min to obtain a homogeneous suspension. A 30 μL aliquot of the suspension was then drop-cast onto the electrode surface and allowed to dry at room temperature. The modified SPEs were characterized using electrochemical impedance spectroscopy (EIS) and cyclic voltammetry (CV), each performed in triplicate (*n* = 3), employing a 5.0 mM solution of [Fe(CN)_6_]^3−^/^4−^ (1 : 1) in 0.1 M KCl as the redox probe and supporting electrolyte. In addition, chronoamperometric measurements were conducted in phosphate-buffered saline (PBS), as shown in Fig. 2.

## 3. Results and discussion

### 3.1 XRD analysis

**Fig. 3:**
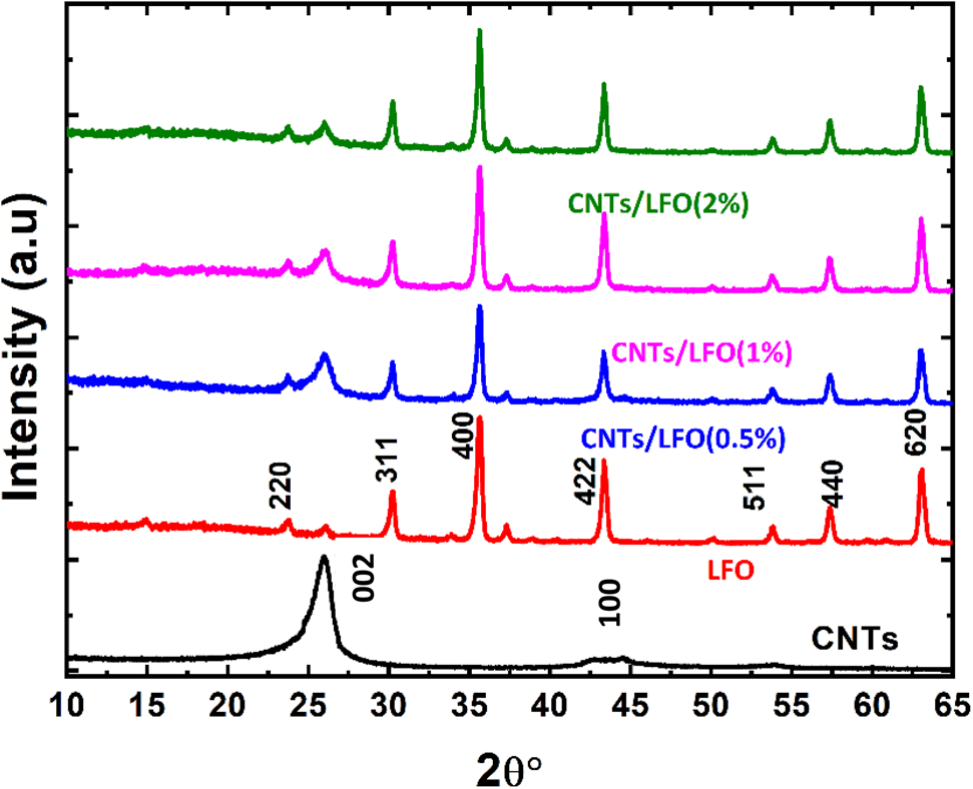
X-ray diffraction (XRD) patterns of the prepared nanocomposite materials: carbon nanotubes (CNTs), lithium ferrite (LFO), and CNTs/LFO nanocomposites.

Structural characterization of the synthesized lithium ferrite nanocomposites was carried out using X-ray diffraction (XRD). Fig. 3 presents the diffractograms of CNTs, pure lithium ferrite, and CNTs incorporated with varying concentrations of LFO. Several diffraction peaks were observed, with the major reflections corresponding to the α-Fe_2_O_3_ phase and the LiFe_5_O_8_ crystal phase. These peaks can be attributed to the interaction between α-Fe_2_O_3_ and free Li^+^ ions.^45,46^

Characteristic peaks at 26.2° and 44.8° for the as-prepared CNTs are corresponding to (002) plane with *d*-spacing reflection of ∼0.33, and (100) plane with *d*-spacing of 0.2 nm respectively.^47,48^ As displayed in Fig. 3, the diffraction peaks of the prepared lithium ferrite nanoparticles located at 23.8°, 30.27°, 35.69°, 43.35°, 53.87°, 57.39 °and 63.01° are attributed to the (220), (311), (400), (422), (511), (440), and (620) planes, respectively, which matches the cubic Li_2_Fe_3_O_5_ (JCPDS card No. 74-1726).^49^ The absence of additional peaks in the XRD pattern revealed that there are no impurity phases and the material is single-phase. Table 1S (SI) shows the crystallite size calculated by the Scherer equation, which matches the determined crystallite size from the SEM image and other calculated parameters.

The crystallinity grade was determined by calculating the area under the crystalline peaks and non-crystalline peaks of the XRD pattern *via* the following equation^50^1
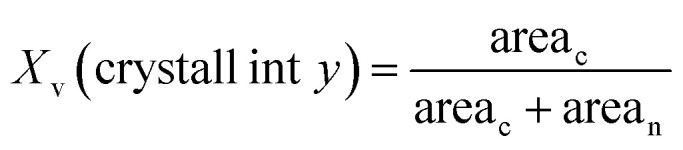
where area_c_ refers to the area of crystalline peaks, and area_n_ represent the non-crystalline peak area.

From Scherrer's equation, the sample crystallite size *D*_ν_ can be valued as:^51^2
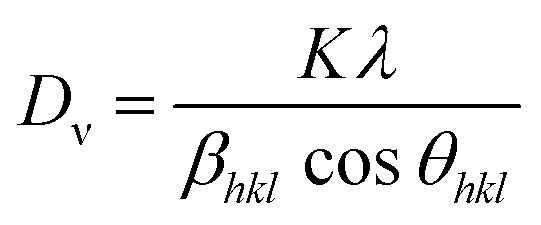
Here, the volume-weighted crystallite size (nm) is *D*_ν_; *k* symbolizes the shape factor (*k* = 0.9), *λ* refers to the wavelength of the X-rays (*λ* = 0.154056 nm for Cu Kα radiation); *θ*_*hkl*_ for Bragg diffraction angle (°) and the broadening of the *hkl* diffraction peak evaluated at half of its maximum intensity (in radians) is *β*_*hkl*_. Further, the quantity of imperfections in the sample, which represents the dislocation density, is determined as (1/*D*_ν_2).^52,53^ The structural parameters are listed in Table 2S (SI).

Alternatively, the two independent factors of lattice strain and crystallite size contribute to the total peak broadening. The strain-induced line broadening *β*_s_ is given by the relation *β*_s_ = 4ε tan θ_*hkl*_.^54^ The sum of the crystallite size and strain in the material can now be determined by the total peak broadening.

**Fig. 4:**
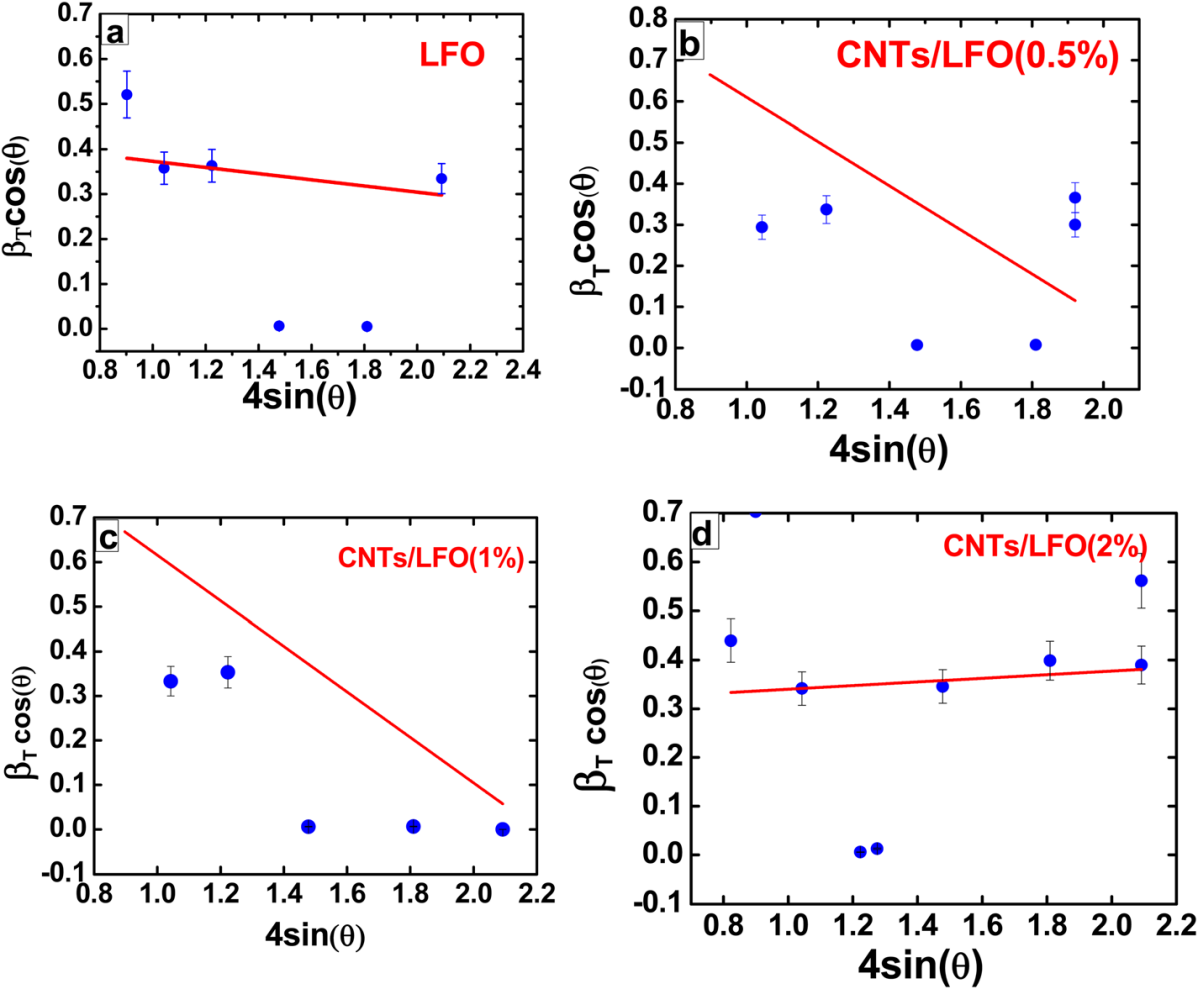
Williamson–Hall (W–H) plots derived from the XRD data for (a) LFO, (b) CNTs/LFO (0.5%), (c) CNTs/LFO (1%), and (d) CNTs/LFO (2%).

The Williamson–Hall (W–H) equation takes into account the isotropic character of the crystal and assumes that the strain in the material is uniform^55^ for the total peak broadening, which can be found using^56^3
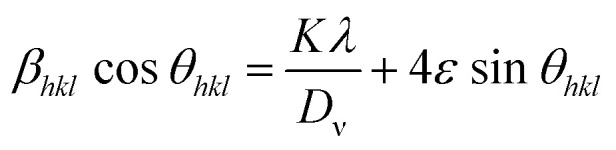
where *k* is the shape factor and *D*_v_ refers to volume-weighted crystallite size. A plot is drawn between 4 sin *θ*_*hkl*_ along the *X*-axis, and *β*_*hkl*_ cos *θ*_*hkl*_ along the *Y*-axis as shown in Fig. 4.

In the Williamson–Hall (W–H) model, the crystallite size is estimated from the intercept of the linear fit, while the slope provides the strain within the material. The values obtained from the W–H plot were further compared with the crystallite size and strain calculated using Scherrer's formula.^57^

### 3.2 Morphological analysis

**Fig. 5:**
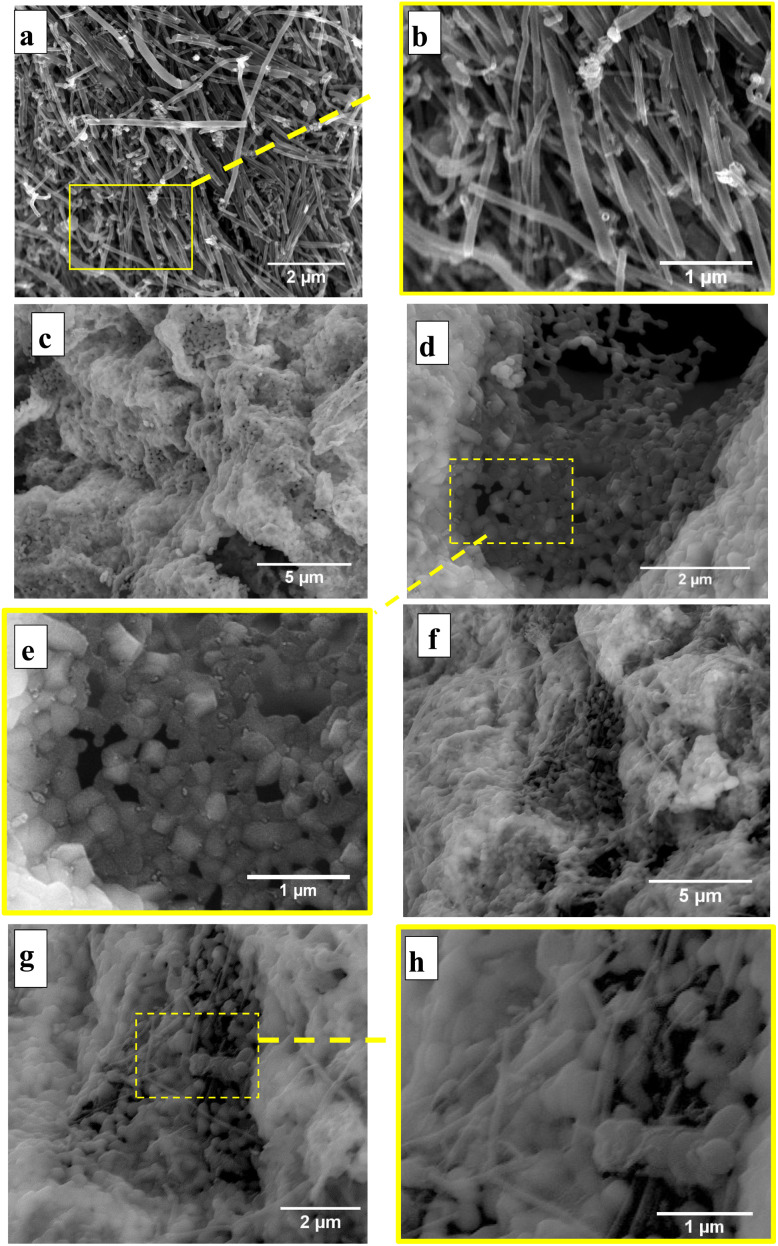
FE-SEM images at different magnifications: (a and b) CNTs, (c–e) LFO, and (f–h) CNTs/LFO (2%).

The morphological features of lithium ferrite (LFO), CNTs/LFO (2%), and carbon nanotubes (CNTs) samples were examined using field emission scanning electron microscopy (FE-SEM), which provides high-resolution nanoscale surface imaging. Fig. 5 presents representative FE-SEM images of LFO, CNTs/LFO (2%), and CNTs at two different magnifications, clearly highlighting their distinct morphological characteristics.

For the CNTs samples, a broad distribution of tube diameters was observed, ranging from ∼44 nm to 243 nm. The measured diameters included 79.55 nm, 122.7 nm, 148 nm, 73.20 nm, 60.33 nm, 64.88 nm, and 74.43 nm. Such variability reflects the complex growth dynamics and aggregation tendencies commonly associated with carbon nanotubes. Morphologically, the CNTs exhibited a characteristic “spaghetti-like” appearance, forming randomly oriented, entangled networks that extended into the micrometer scale. The tubes were not uniformly straight but were loosely interconnected, consistent with the aggregation behavior reported in previous CNTs studies.^48,58^

In contrast, the FE-SEM images of pure lithium ferrite (LFO) unveiled a distinctive raspberry-like morphology. This was characterized by the noticeable aggregation of spherical grains, which were the predominant feature observed in the sample. While these grains were primarily spherical, they also displayed prismatic characteristics, highlighting the complexity of the LFO structure.^59,60^

**Fig. 6:**
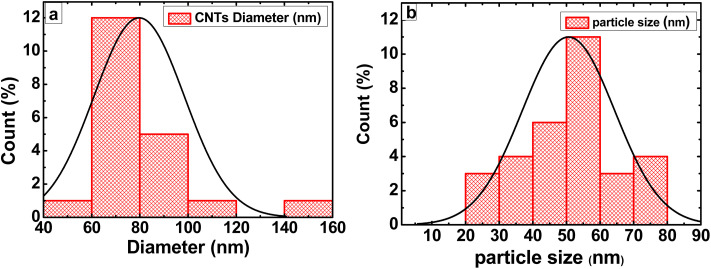
Histograms of the diameter and particle size distributions for (a) CNTs and (b) lithium ferrite (LFO).

The diameter distribution of the CNTs/LFO nanocomposite was further analyzed using the ImageJ software to provide insights into the size characteristics of the synthesized nanomaterials. As shown in Fig. 6(a), the CNTs exhibited an average diameter of ∼80 nm, while the LFO nanoparticles displayed an average size of ∼50 nm, as presented in Fig. 6(b). These findings confirm the successful synthesis of CNTs/LFO nanocomposites with well-defined nanostructured features. The nanoscale integration of CNTs and LFO is anticipated to enhance the overall performance of the composite, particularly in electrochemical sensing applications, owing to their complementary morphologies and the synergistic interactions at the CNTs-LFO interface.

### 3.3 Magnetism of the synthesized samples

**Fig. 7:**
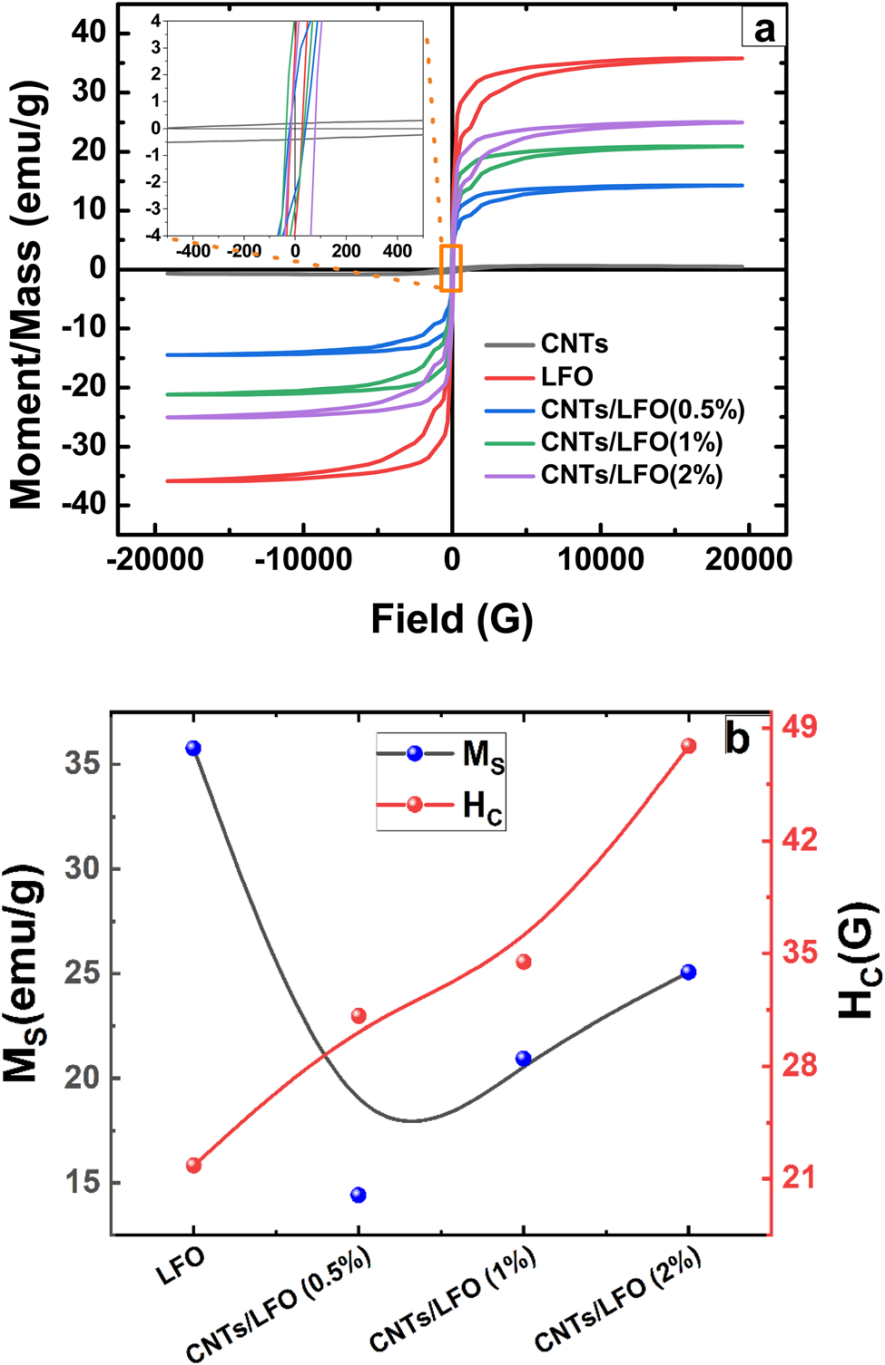
Magnetic measurements obtained using vibrating sample magnetometry (VSM): (a) hysteresis loops of pure CNTs and CNTs/LiFe_5_O_8_ nanocomposites recorded at room temperature over an applied magnetic field range of ±20 kG, and (b) comparison of the saturation magnetization and coercivity values of CNTs/LiFe_5_O_8_ nanocomposites with those of pristine lithium ferrite.

Magnetic properties of the synthesized samples, with and without lithium ferrite doping, were investigated using vibrating sample magnetometry (VSM). Fig. 7 shows the corresponding hysteresis loops obtained over a wide range of externally applied magnetic fields. The shape of the hysteresis loop provides critical information for evaluating the magnetic behavior of the materials. A wide hysteresis loop, typically associated with large coercivity, is characteristic of hard magnetic materials, which are suitable for applications such as magnetic recording and transformers. In contrast, a narrow hysteresis loop with low coercivity corresponds to soft magnetic materials, making them more suitable for biomedical and hyperthermia applications. The observed ferromagnetic behavior arises from the unequal orientation of atomic magnetic moments within the material.^61,62^ Moreover, the magnetic features are connected and influenced by the degree of crystallinity and morphological specifications.^63^ The ferromagnetic behavior is noticed in the samples. The CNTs have a limited saturation amount in comparison to other ferrites and exhibit the lowest value among the delivered samples. These values are close to those in previous reports.^37^

Lithium ferrite exhibited the highest magnitude of saturation, reaching ∼35 emu g^−1^ at 2 kG and sustaining a plateau afterward (refer to Fig. 7 for the M–H loop), which were in agreement with previous results.^64,65^ Upon the inclusion of lithium ferrites, the saturation increases in a portion almost comparable to the concentration of the doped lithium ferrite nanoparticles. Possible reasons for the saturation elevation might be attributed to the gradual upsurge of crystallinity or particle size.^66^ In addition, the reduction in the coercivity magnitude is predicted as per the soft nature of lithium ferrite and might be ascribed to the surface anisotropy or the potential tendency and crossover of the multiphase domain shift, as clarified by the reduction in the squareness value.^66^ The squareness value determined from the saturation and remanent magnetization amounts, discriminates between the single-phase state with uniaxial magnetic anisotropy, and the multi-domain state. The enhanced magnitude of 0.5 or higher reflects the occurrence of a single domain phase (close to 0.5).^34,67^ When the value deviates and becomes lower than 0.5 it designates the multi-domain state. The obtained values are depicted in Table 3S (SI). The raised saturation and the lowered coercivity magnitudes affirm the decline in the progress of magnetic anisotropy, allowing the alignment of the moment in the applied field.^67^

The coercivity magnitude emerges as the average of the right and left intersection points of the hysteresis loop branches with the *x*-axis, mathematically expressed as^68^4
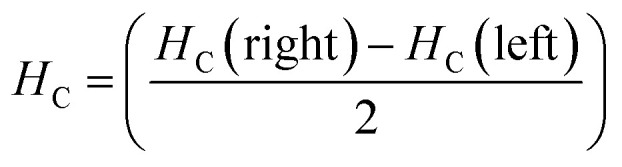


### 3.4 Electrochemical characteristics of CNTs–LiFe_2_O_3_ nanocomposites

Screen-printed electrodes (SPEs) were employed as substrates to evaluate the electrochemical properties of the synthesized nanomaterials using cyclic voltammetry (CV) and electrochemical impedance spectroscopy (EIS). Both bare SPEs and nanocomposite-modified SPEs were tested in a standard redox solution of 5 mM [Fe(CN)_6_]^3−^/^4−^ (1 : 1) containing 0.1 M KCl as the supporting electrolyte.

**Fig. 8:**
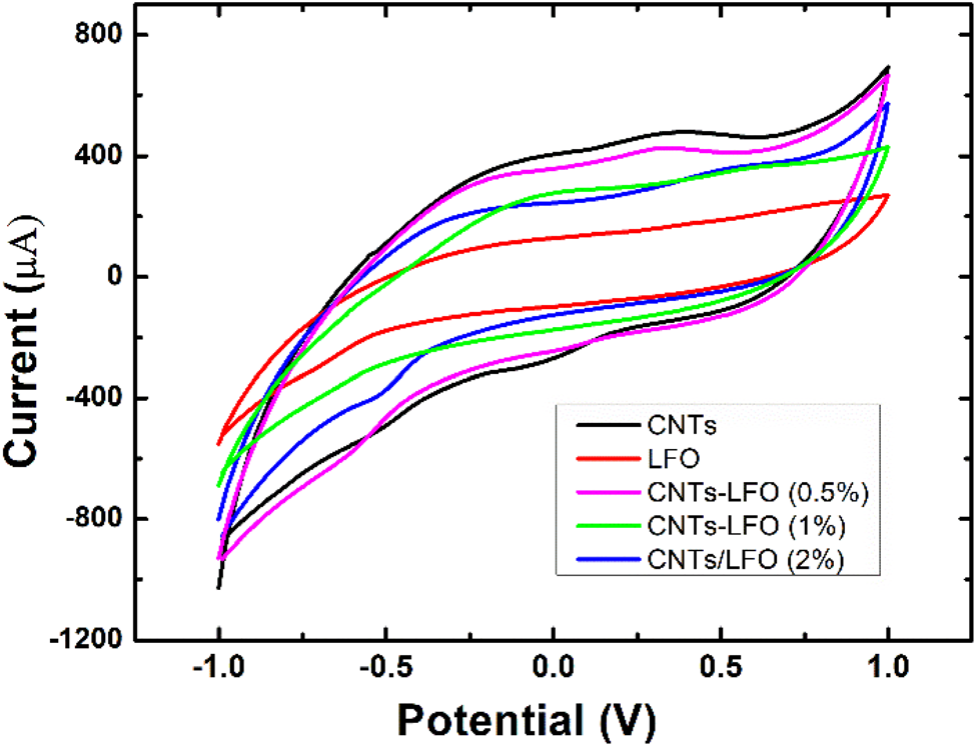
Cyclic voltammetry (CV) responses of SPEs modified with CNTs, lithium ferrite (LFO), and CNTs/LFO nanocomposites. Measurements were recorded in 5 mM [Fe(CN)_6_]^3−^/^4−^ (1 : 1) containing 0.1 M KCl by scanning the potential from −1.0 to +1.0 V at a scan rate of 50 mV s^−1^.

**Fig. 9:**
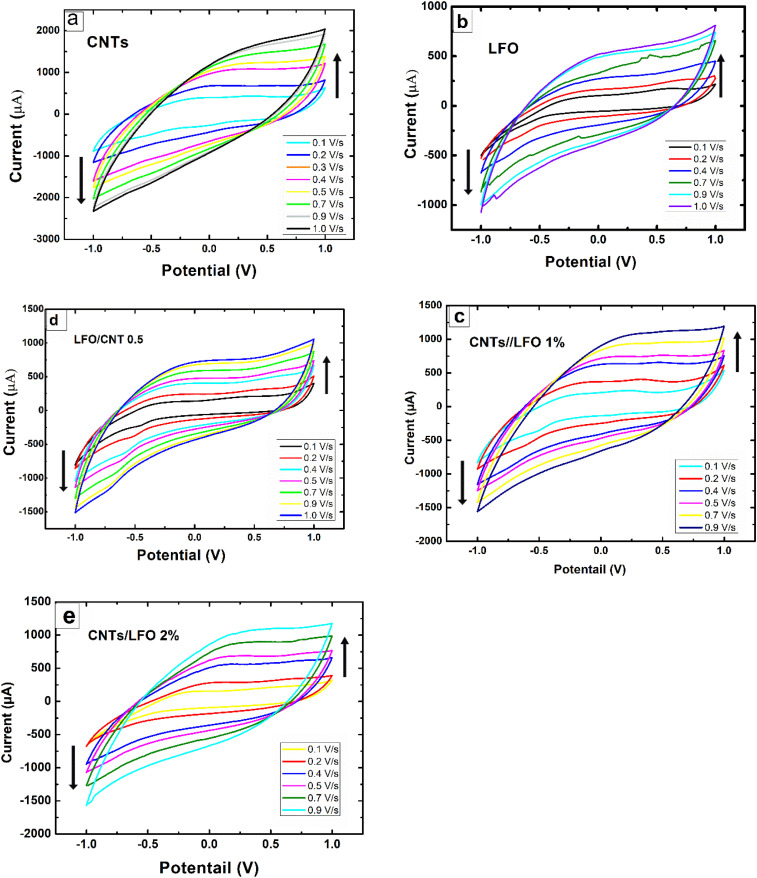
Cyclic voltammetry (CV) of SPEs modified with the prepared nanomaterials ((a) CNTs, (b) LFO, and (c–e) CNTs/LFO nanocomposites) at different scan rates, illustrating the effect of scan rate on the electrochemical response.

Fig. 8 displays the voltammetric responses of the prepared nanomaterials. All modified electrodes exhibited higher faradaic currents compared to the bare SPE, indicating faster electron transfer kinetics for the standard redox probe. The effect of scan rate on the electrochemical response of each sample was further investigated by CV (Fig. 9). The incorporation of ferrite into CNTs significantly enhanced the charge transfer characteristics of the composite-modified electrodes, with the 0.5% LFO concentration providing the greatest improvement.

**Fig. 10:**
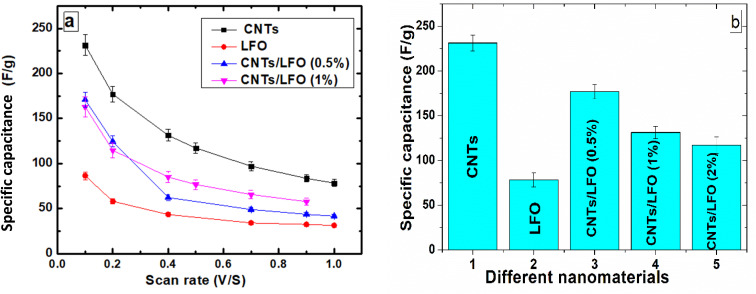
(a) Specific capacitance of CNTs, LFO, and CNTs/LFO nanocomposites at different scan rates. (b) Histogram comparing the calculated specific capacitance values of the different prepared samples.

To gain further insights, the influence of scan rate on the specific capacitance of each electrode was evaluated (Fig. 10(a)). Pure LFO exhibited very low capacitance values compared to CNTs, whereas the CNTs/LFO nanocomposites demonstrated substantial enhancements across different scan rates, highlighting their suitability for electrochemical applications (Fig. 10(b)).

**Fig. 11:**
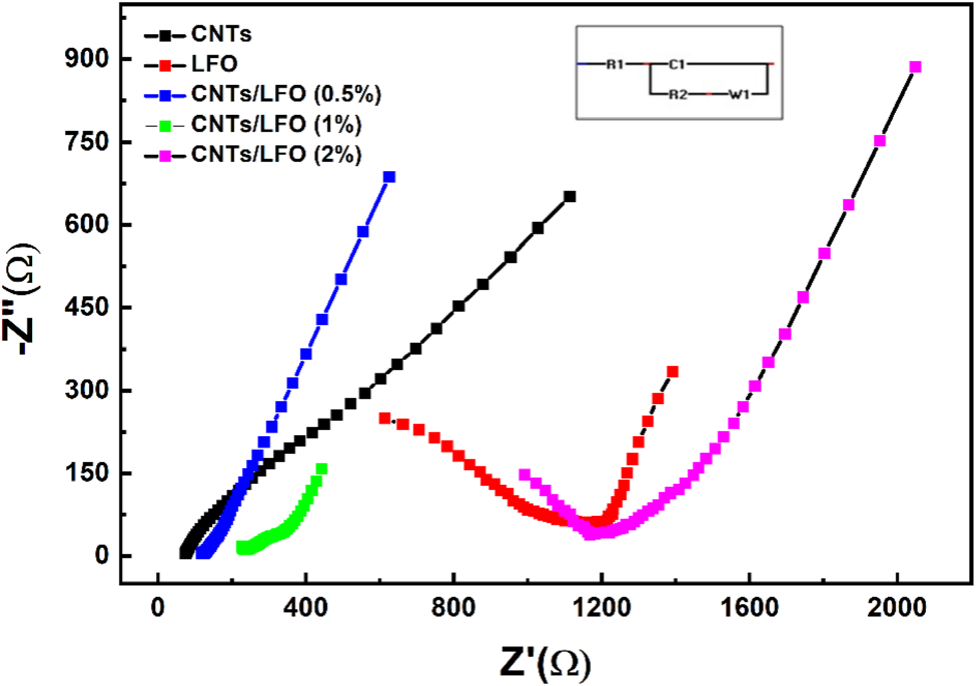
Electrochemical impedance spectroscopy (EIS) spectra (Nyquist plots) of CNTs, LFO, and CNTs/LFO nanocomposites, showing imaginary impedance (–*Z*ʺ) *versus* real impedance (*Z*′). Measurements were performed at an open-circuit potential with a 10 mV AC amplitude over the frequency range 10 000–0.1 Hz. The spectra were fitted to an equivalent circuit model to extract key parameters such as charge transfer resistance (*R*_ct_).

EIS spectra, represented by Nyquist plots (imaginary impedance, −*Z*ʺ, *versus* real impedance, *Z*′), are shown in Fig. 11. The CNTs-modified electrode exhibited a markedly lower charge transfer resistance (*R*_ct_ = 80.5 Ω) compared to the electrodes without CNTs, reflecting improved electron transfer facilitated by the highly conductive CNTs framework. Among the composites, CNTs/LFO (0.5%) displayed the lowest *R*_ct_ value (50.3 Ω), outperforming CNTs/LFO (1%) (*R*_ct_ = 205.9 Ω). In contrast, pure LFO electrodes showed the highest resistance (*R*_ct_ = 1150.3 Ω). These findings confirm the superior charge transfer properties of the CNTs/LFO (0.5%) composite relative to other formulations.

The quantitative electrochemical parameters, including anodic potential (*E*_a_), cathodic potential (*E*_c_), peak-to-peak separation (Δ*E*), anodic current (*I*_a_), cathodic current (*I*_c_), charge transfer resistance (*R*_ct_), constant phase element (CPE), and Warburg impedance (W), are summarized in Table S4 (SI).

### 3.5 Functionalization of Li_2_Fe_3_O_5_-based carbon nanocomposites for H_2_O_2_ detection

Recent studies have highlighted various strategies for improving hydrogen peroxide (H_2_O_2_) detection and production through the rational design of semiconductor-based composites. For example, Li *et al.* (2025) reported the fabrication of an S-scheme W_18_O_49_/ZnIn_2_S_4_ photocatalyst, which demonstrated enhanced H_2_O_2_ generation due to the efficient charge separation and transfer pathways created within the heterojunction structure. Such advances underscore the importance of nanocomposite engineering, where tailored interfaces and synergistic effects can significantly improve electrochemical and photocatalytic performance.

In this context, we focus on the functionalization of Li_2_Fe_3_O_5_-based carbon nanocomposites as a promising approach for non-enzymatic H_2_O_2_ detection. The integration of carbon nanotubes with ferrite nanoparticles is expected to facilitate rapid electron transfer, enhance conductivity, and provide abundant active sites, thereby improving sensitivity and lowering detection limits in electrochemical sensing applications.^27^ Du *et al.* (2025) further demonstrated the role of H_2_O_2_ as a reactive mediator in photocatalytic pollutant degradation using Bi-based composites. Their work provided valuable insights into how catalytic nanocomposites interact with H_2_O_2_, particularly in accelerating redox processes and improving degradation efficiency. These findings emphasize the broader versatility of H_2_O_2_ not only as a target analyte for sensing applications but also as a functional species in advanced catalytic systems.

Drawing inspiration from these studies, the functionalization of Li_2_Fe_3_O_5_-based carbon nanocomposites offers a dual advantage: (i) efficient electron transfer enabled by the conductive CNTs network, and (ii) catalytic activity stemming from ferrite nanoparticles that actively interact with H_2_O_2_ molecules. This synergy positions the CNTs/Li_2_Fe_3_O_5_ composites as highly promising candidates for sensitive, reliable, and enzyme-free H_2_O_2_ detection.^28^

Furthermore, recent studies, such as those by Guo *et al.*, have emphasized the critical role of microstructure regulation in CNTs-semiconductor composites. Their findings demonstrated that tailoring the interfacial architecture can markedly enhance electron transfer pathways and catalytic activity. This concept is highly relevant to the design rationale of CNTs/LFO nanocomposites, where the controlled integration of lithium ferrite with the CNTs network is expected to optimize interfacial charge dynamics and, consequently, improve sensing performance.^26^

**Fig. 12:**
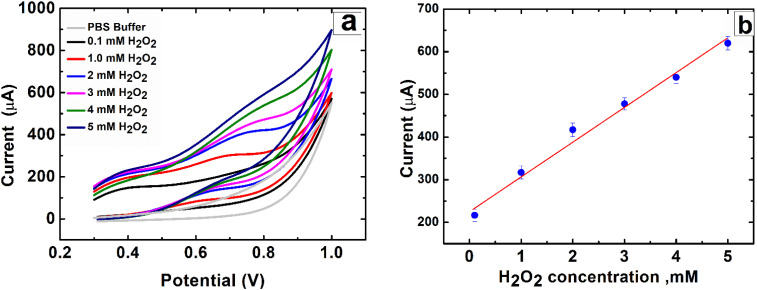
(a) Cyclic voltammograms recorded for CNTs/LFO-modified SPEs in the presence of varying H_2_O_2_ concentrations, using phosphate-buffered saline (PBS, pH = 7.4) as the supporting electrolyte. (b) Corresponding calibration curve of the current response *versus* H_2_O_2_ concentration, demonstrating the electrocatalytic performance of the CNTs/LFO electrode.

The primary objective of this research is to engineer materials capable of achieving rapid and direct electron transfer while exhibiting superior electrochemical performance for sensing applications. In this context, carbon nanotubes (CNTs) and metal oxides were systematically evaluated for their ability to promote the direct oxidation of hydrogen peroxide (H_2_O_2_). Cyclic voltammetry (CV) measurements were performed by introducing different concentrations of H_2_O_2_ into the electrochemical cell to assess the feasibility of direct oxidation. As shown in Fig. 12(a), well-defined oxidation peak currents for H_2_O_2_ appeared at approximately 0.7 V without the need for artificial redox mediators, confirming the synergistic contribution of the modified electrodes. Notably, the CNTs/LFO (0.5%) nanocomposite-modified electrode exhibited the highest electrocatalytic activity, enabling efficient and direct detection of H_2_O_2_ (Fig. 12(b)).

#### 3.5.1 Influence of pH

**Fig. 13:**
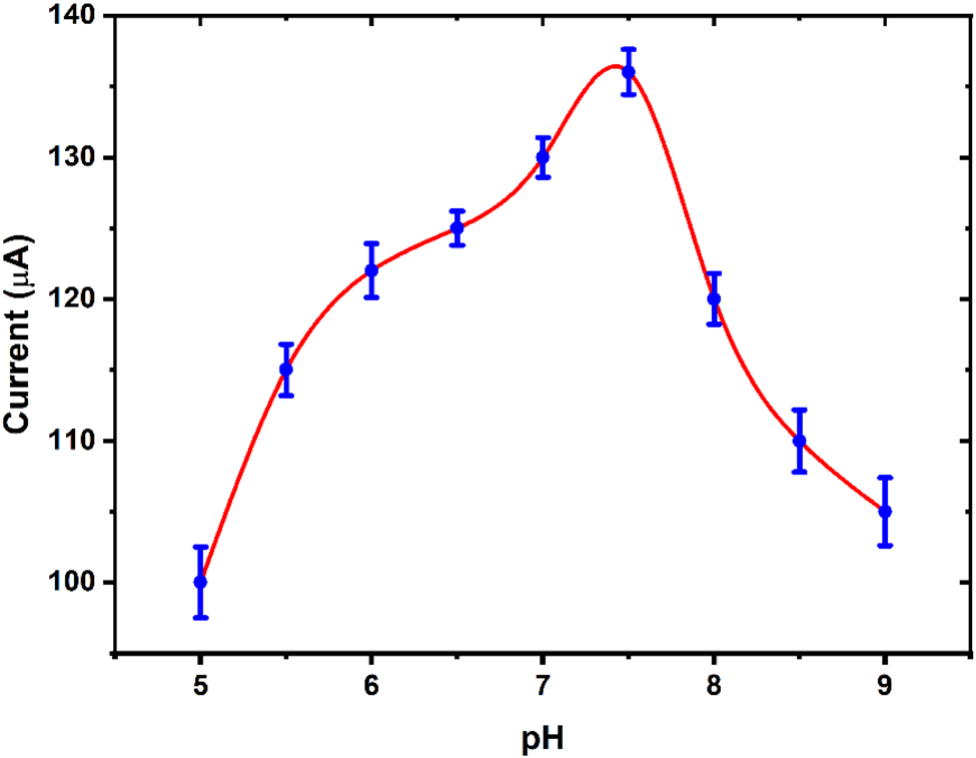
Effect of pH on hydrogen peroxide oxidation current using CNTs/LFO-modified screen-printed electrodes (SPEs). Chronoamperometric measurements were performed at a fixed potential of 0.7 V.

The effect of pH on the electrochemical oxidation of H_2_O_2_ at CNTs/LFO-modified electrodes was systematically investigated. As shown in Fig. 13, the oxidation current increased with rising pH, reaching its maximum at pH 7.4, where the highest current response was observed. Beyond this optimum value, the current gradually decreased. The reduction in oxidation current at acidic conditions can be attributed to protonation of the electrode surface, which suppresses electron transfer at the electrode/electrolyte interface. Conversely, at higher pH values, the diminished current may result from the reduced availability of protons, which slows down the oxidation process. Thus, pH 7.4 was identified as the optimal condition for efficient H_2_O_2_ detection using the CNTs/LFO nanocomposite electrode.^16^ From the obtained results, pH 7.4 was identified as the optimal value for subsequent experiments. This finding is particularly advantageous, as the physiological pH of 7.4 coincides with the optimum sensing condition. Consequently, the developed sensor not only achieves superior electrochemical performance but also operates effectively under biologically relevant environments, underscoring its potential for practical biomedical applications.

#### 3.5.2 Peroxide detection

The capability of the nanocomposite-modified electrodes to directly oxidize hydrogen peroxide (H_2_O_2_) was first confirmed by cyclic voltammetry. Based on these findings, chronoamperometric measurements were subsequently performed at an applied potential of 0.7 V. Incremental additions of H_2_O_2_ were introduced at fixed time intervals following a standard addition protocol.

**Fig. 14:**
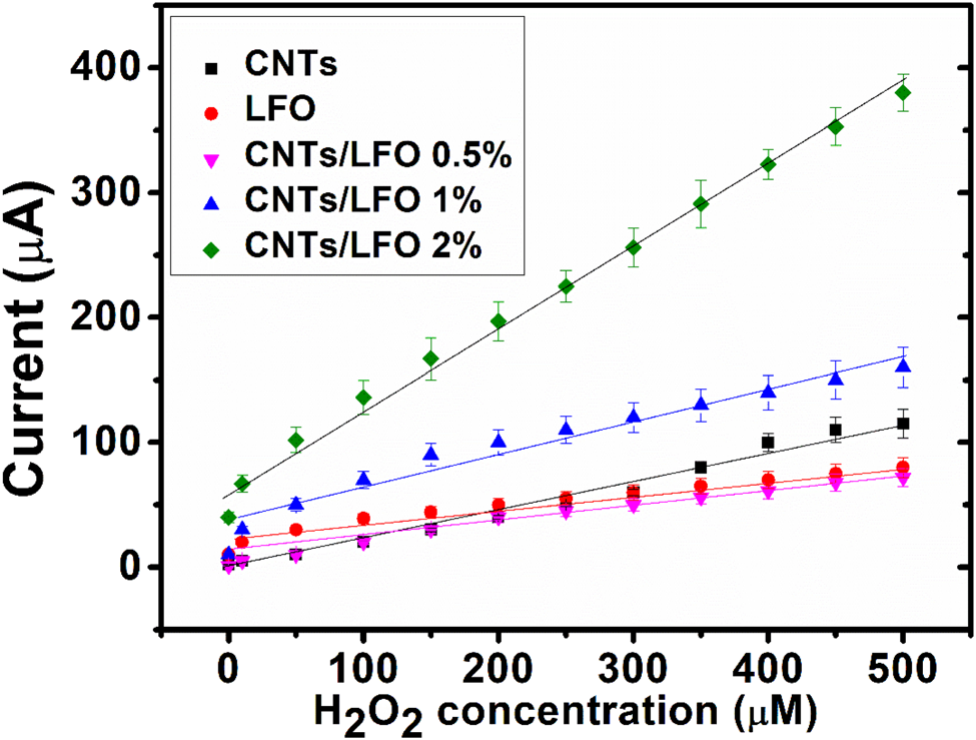
Amperometric responses of CNTs, LFO, CNTs/LFO (0.5%), CNTs/LFO (1%), and CNTs/LFO (2%) electrodes toward successive additions of H_2_O_2_ at 0.7 V in PBS (pH 7.4).

As shown in Fig. 14, the resulting calibration curves illustrate the amperometric responses of different electrodes (CNTs, LFO, CNTs/LFO (0.5%), CNTs/LFO (1%), and CNTs/LFO (2%)) across a range of H_2_O_2_ concentrations in PBS buffer (pH = 7.4). Among all tested electrodes, the CNTs/LFO (1%) nanocomposite delivered the most pronounced current response for each peroxide addition. This superior performance is attributed to the synergistic interaction between CNTs and LFO, which enhances charge transfer and electrocatalytic activity, thereby facilitating more efficient direct oxidation of H_2_O_2_.

A comparative evaluation of the detection potential, detection limit, and linear range of CNTs/LFO (1%) with other reported H_2_O_2_ sensors is summarized in Table 5S (SI). The analytical performance of CNTs/LFO (1%) is comparable to, and in several aspects superior to, many previously developed electrodes. Owing to its low detection limit and wide linear range, CNTs/LFO (1%) emerges as a promising sensing material for H_2_O_2_ detection, making it suitable for a broad spectrum of sensing and biosensing applications.

The obtained voltammetric and chronoamperometric results confirmed the capability of the nanocomposite-based SPE to directly oxidize hydrogen peroxide. To further validate the sensor's analytical performance, square wave voltammetry (SWV), differential pulse voltammetry (DPV), and chronoamperometry (CA) were employed using the same CNTs/LFO (1%) modified electrode.

**Fig. 15:**
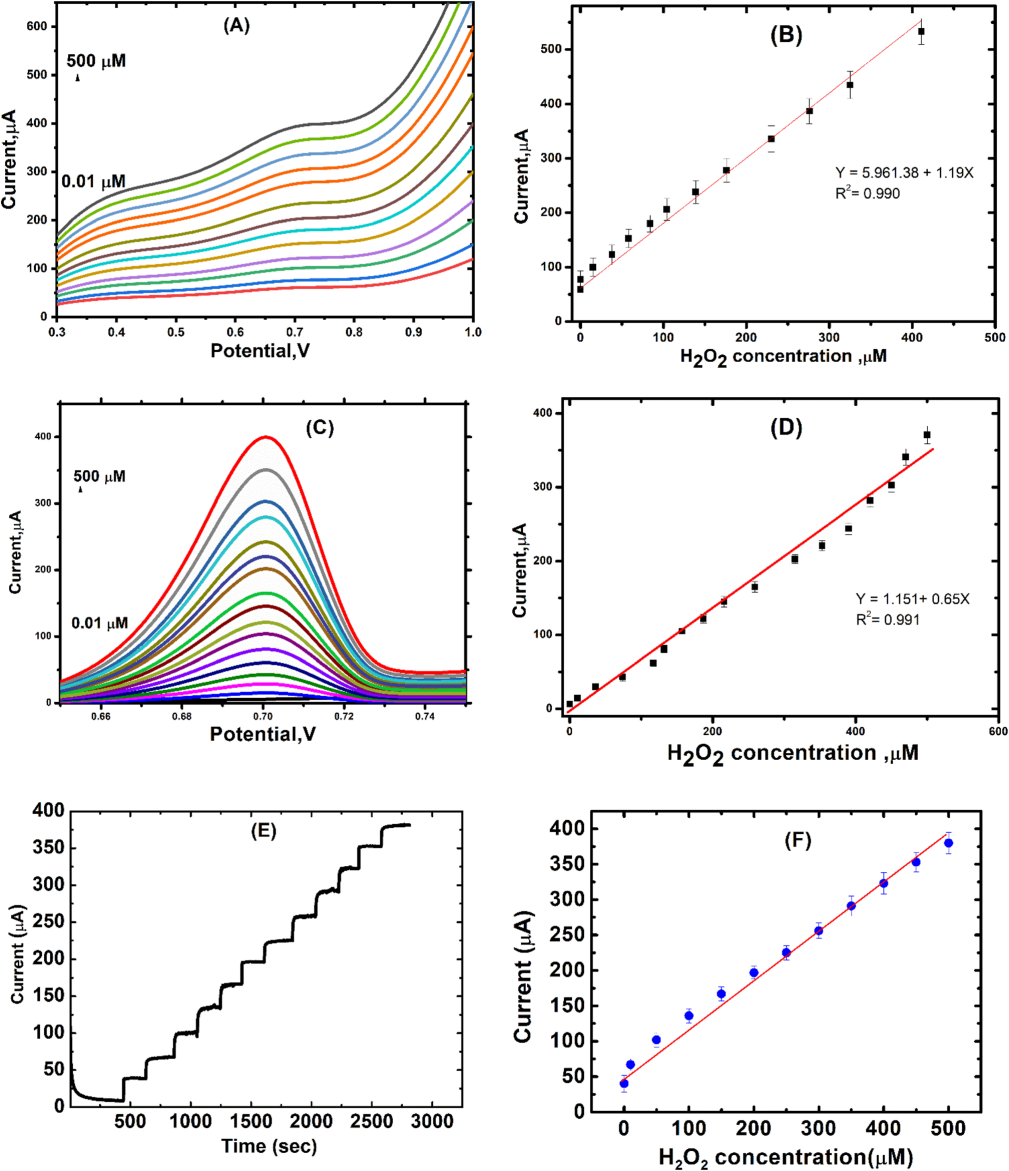
Electrochemical responses and corresponding calibration curves obtained by SWV (A and B), DPV (C and D), and CA (E and F) for hydrogen peroxide detection using the CNTs/LFO (1%) nanocomposite-modified electrode in PBS (pH 7.4) at 0.7 V.

The SWV results (Fig. 15a and b) depicted the SWV responses and the corresponding calibration curves at varying H_2_O_2_ concentrations. The sensor demonstrated a sensitivity of 1.19 μA μM^−1^ ± 0.08, a wide linear detection range from 0.01 to 500 μM, excellent linearity (*R*^2^ = 0.997), a very low detection limit (LOD = 0.005 μM, S/N = 3), and a quantification limit (LOQ = 0.07 μM).

The DPV results shown in Fig. 15c and d revealed a sensitivity of 0.651 μA μM^−1^ ± 0.03, with a broad linear detection range from 0.01 to 500 μM and high correlation (*R*^2^ = 0.9975). The calculated LOD and LOQ were 0.03 μM (S/N = 3) and 0.05 μM, respectively. The CA results (presented in Fig. 15e and f) exhibited a sensitivity of 0.651 μA μM^−1^ ± 0.02, a linear range of 0.1 to 500 μM, with *R*^2^ = 0.991. The LOD and LOQ were found to be 0.008 μM (S/N = 3) and 0.08 μM, respectively.

When benchmarked against peroxide sensors reported in the literature (Table 5S, SI), the CNTs/LFO (1%) electrode clearly outperforms many existing systems in terms of sensitivity, detection limit, and linear working range, underscoring its strong potential for high-performance electrochemical sensing applications.

This table highlights the superior performance of the CNTs/LFO-modified electrode, which achieves an exceptionally high sensitivity, selectivity, a low detection limit of 0.005 μM and a broad linear detection range spanning 0.01–500 μM. Compared to other reported electrode materials, the CNTs/LFO sensor demonstrates significant advantages in sensitivity and range. For instance, CNT-NiCo_2_O_4_-modified SPEs exhibited a linear range of 2.5–275 μM with a detection limit of 0.01 μM,^69^ while NiCo_2_O_4_/RGO-modified GCEs achieved a range of 5–3000 μM with a detection limit of 0.41 μM.^70^ Similarly, MnCo_2_O_3_/CNTs/SPEs reported a linear range of 0.1–180 μM and a detection limit of 0.1 μM.^14^ The rGO-Pt/GCE sensor offered a wider range^71^ of 0.5–3475 μM but a higher detection limit of 0.2 μM,^7^ whereas Co_3_O_4_/SPEs demonstrated a narrower range of 0.1–50 μM with a detection limit of 0.145 μM.^72^ The CoFe_2_O_4_/CNTs/GCE sensor exhibited a linear range of 0.5–50 μM and a detection limit of 0.05 μM.^43^ Additionally, Pt/rGO-CNT exhibited a range of 0.1–25 μM with a detection limit of 0.1 μM,^73^ and Pol(azure A)-PtNPs/SPEs displayed a range of 0–300 μM with a detection limit of 0.052 μM.^20^ In contrast, Co_3_O_4_-rGO composites offered a wider range of 15–675 μM but with a considerably higher detection limit of 2.4 μM.^74^

### 3.6 Selectivity

**Fig. 16:**
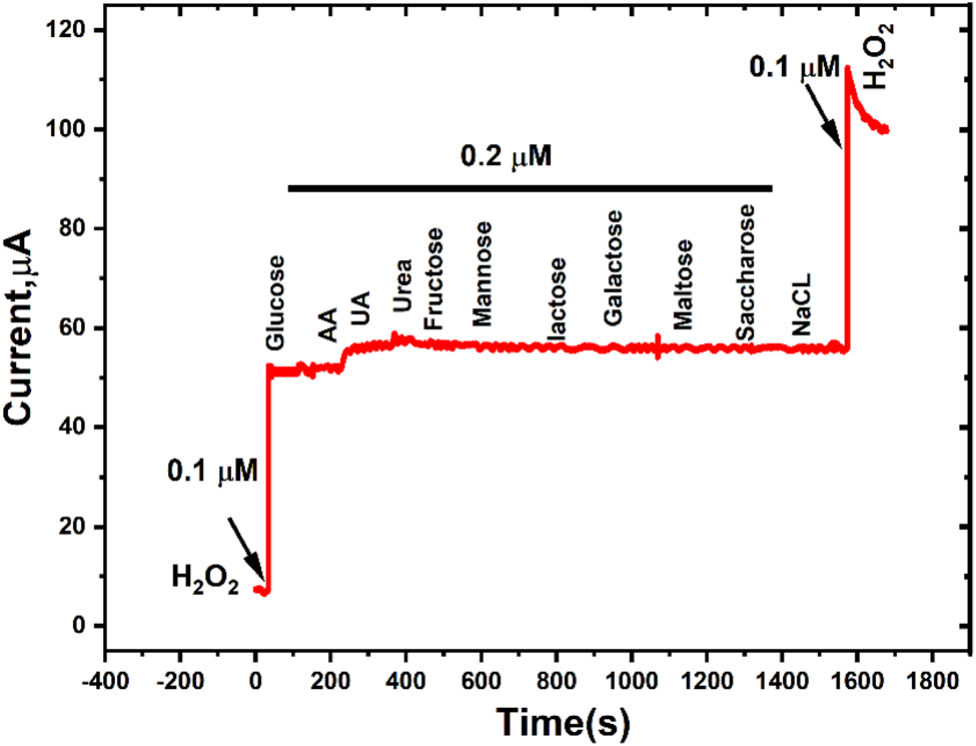
Chronoamperometric responses of the CNTs/LFO-modified electrode to sequential additions of 0.1 μM H_2_O_2_ and 0.2 μM of common potential interferents, including ascorbic acid (AA), uric acid (UA), urea, fructose, mannose, lactose, maltose, sucrose, NaCl, and glucose.

The selectivity of the CNTs/LFO-modified sensor toward hydrogen peroxide detection was investigated by recording its electrochemical response in the presence of 0.1 μM H_2_O_2_ alongside common potential interferents at two-fold higher concentrations (0.2 μM). The tested species included ascorbic acid (AA), uric acid (UA), urea, fructose, mannose, lactose, maltose, sucrose, NaCl, and glucose. As shown in Fig. 16, the chronoamperometric signal displayed a pronounced increase exclusively upon the addition of H_2_O_2_, while negligible or no current variations were observed with the interfering compounds. These findings confirm the high selectivity of the proposed sensor for peroxide detection under the studied conditions.

### 3.7 Stability, repeatability, and reproducibility

The electrochemical stability of the non-enzymatic CNTs/LFO sensor was investigated over 500 consecutive CV cycles in H_2_O_2_ solution, during which the peak current retained over 92% of its initial value, indicating excellent electrochemical stability. The amperometric response of the sensor to a fixed concentration of H_2_O_2_ (0.1 μM) was monitored over a period of one month under ambient storage conditions. The response showed only a minor decrease (less than 5.6%) in the current signal, demonstrating good durability and repeatability.

**Fig. 17:**
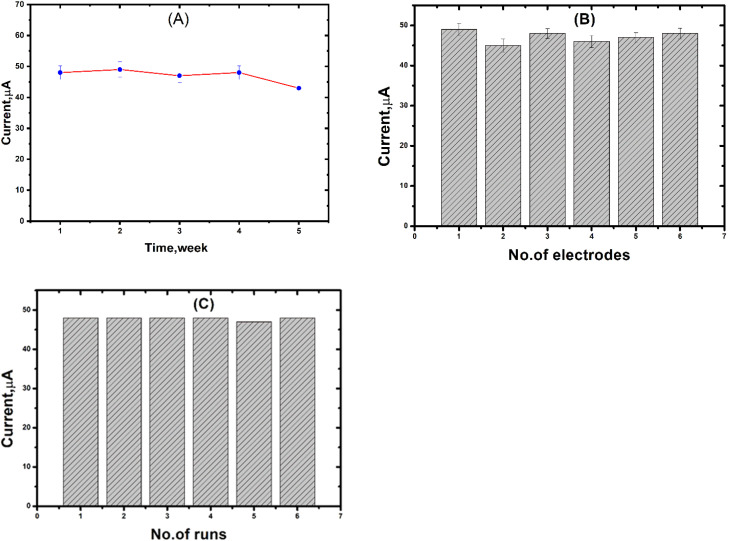
(A) Stability of the CNTs/LFO-modified electrode toward 0.1 μM H_2_O_2_. (B) Reproducibility of the sensor response using six independently prepared electrodes. (C) Repeatability of the electrode fabrication process under identical conditions. All measurements were conducted using sequential injections of 0.1 μM H_2_O_2_.

As illustrated in Fig. 17(a), the oxidation current after 30 days retained 86.87% of its initial value, indicating strong stability under repeated use. Repeatability was evaluated by testing the same electrode six times under identical conditions (Fig. 17(b)), resulting in a relative standard deviation (RSD) of 1.09%, which reflects excellent short-term measurement consistency. Reproducibility was assessed using six separately fabricated electrodes tested under the same conditions with 2 mM peroxide (Fig. 17(c)). The calculated RSD of 2.36% confirms the reliability and uniformity of the electrode fabrication process.

### 3.8 Real sample analysis

The practical applicability of the proposed non-enzymatic CNTs/LFO-based peroxide sensor was validated using wastewater samples, with the results summarized in Table 6S (SI). The relative standard deviation (RSD) values, ranging from 0.192% to 2.96%, demonstrated the high precision and reproducibility of the sensor in complex real matrices. Moreover, the peroxide concentrations determined by the sensor were in close agreement with the spiked values, confirming its accuracy and reliability. These results indicate that the sensor maintains excellent performance not only in standard peroxide solutions but also in real sample environments. Hence, the CNTs/LFO sensor exhibits strong potential for practical applications in environmental monitoring, clinical diagnostics, and other biosensing fields.

## 4. Conclusion

In this study, lithium ferrite (Li_2_Fe_3_O_5_; LFO) and carbon nanotube (CNT) nanocomposites were successfully synthesized and characterized at varying LFO concentrations to produce CNTs/LFO hybrids. XRD confirmed the successful formation of the desired crystalline structure, while morphological analysis revealed distinct features: CNTs displayed a spaghetti-like morphology with variable diameters, whereas LFO exhibited a raspberry-like structure composed of spherical and prismatic grains. Magnetic characterization by vibrating sample magnetometry showed that pristine lithium ferrite achieved the highest saturation magnetization (∼35 emu g^−1^), while CNTs displayed limited magnetic response. The enhancement of saturation magnetization upon the incorporation of LFO was attributed to improved crystallinity and particle size, while the reduced coercivity of the composites indicated favorable soft magnetic behavior and a trend toward single-domain characteristics.

Electrochemical evaluation using screen-printed electrodes (SPEs) through cyclic voltammetry and electrochemical impedance spectroscopy revealed significant performance improvements upon integrating CNTs into the LFO matrix. The nanocomposites demonstrated enhanced faradaic currents, superior charge transfer, and excellent specific capacitance with long-term stability, nominating them as promising candidates for electrochemical applications, including sensors and energy storage devices.

Importantly, the CNTs/LFO nanocomposite electrode exhibited excellent potential for non-enzymatic hydrogen peroxide (H_2_O_2_) sensing, delivering high sensitivity, broad linear range, remarkably low detection limits, and strong compatibility with physiological conditions. These advantages were confirmed using square wave voltammetry (SWV), differential pulse voltammetry (DPV), and chronoamperometry (CA).

Despite these promising results, certain limitations remain. The long-term operational stability of the sensor in complex biological or industrial matrices requires further investigation. Additionally, optimization of material ratios and surface functionalization strategies may further improve selectivity under real-world conditions.

Overall, this study demonstrates that CNTs/LFO nanocomposites combine excellent magnetic and electrochemical properties, positioning them as versatile candidates for advanced applications in catalysis, magnetic recording, energy storage, and particularly electrochemical sensing. The findings lay a strong foundation for future optimization and real-world deployment of CNTs/LFO-based sensors as cost-effective, reliable, and high-performance alternatives to conventional peroxide detection systems.

## Author contributions

E. Ouda initiated the research concept, analyzed the experimental results, and contributed to data interpretation and drafting of the manuscript. N. Yousf participated in the experimental work, data analysis, interpretation, and manuscript preparation. A. Elzwawy contributed to draft writing, interpretation of results, manuscript revision, and validation of the experimental process. H. Magar and R. Y. A. Hassan were involved in electrochemical data acquisition, result interpretation, and manuscript writing. M. El-Ashry conceptualized the study, approved the methodology, and validated the findings. E. M. Duraia supervised the research, coordinated the project, contributed to manuscript preparation, clarified data, and ensured the overall integrity of the work. All authors discussed the results, contributed to the final version of the manuscript, and approved its submission.

## Conflicts of interest

The authors announce that they have no conflicts to address.

## Supplementary Material

RA-015-D5RA04502A-s001

## Data Availability

All the acquired data and results for this work are included in the manuscript and the SI. Supplementary information is available. See DOI: https://doi.org/10.1039/d5ra04502a.
