# Supplementary material for: Carbon nanotubes/lithium ferrite nanocomposites: magnetic and electrochemical optimization for enhanced H2O2 sensing
Source: RSC Adv. 2025 Sep 15;15(40):33667–81. doi: 10.1039/d5ra04502a (PMC12435571; doi:10.1039/d5ra04502a)
Supplement: RA-015-D5RA04502A-s001 [file RA-015-D5RA04502A-s001.pdf]

Supplementary Information (SI) for RSC Advances.
This journal is © The Royal Society of Chemistry 2025

Carbon Nanotube/Lithium Ferrite Nanocomposite: Magnetic and Electrochemical Optimization for Enhanced H_2O_2 Sensing

Emtinan Ouda[1], Nehad Yousf[1,5], Amir Elzwawy[2*], Hend S. Magar[3], Rabeay Y. A. Hassan[4], Magdy El-Ashry[1], and El-Shazly M. Duraia[1]

[1]*Physics Department, Faculty of Science, Suez Canal University, Ismailia 41522, Egypt.*
[2]*Ceramics Department, Advanced Materials Technology and Mineral Resources Research Institute, National Research Centre (NRC), 33 El Bohouth St., Dokki, Giza, 12622, Egypt.*

[3]*Applied Organic Chemistry Department, National Research Centre (NRC), 33 El Bohouth St., Dokki, Giza, 12622, Egypt.*

[4]*Biosensors Research Lab, Zewail City of Science and Technology, 6th October City, Giza 12578, Egypt*
[5] *Laboratories Manager, Faculty of Medicine, Suez Canal University, Ismailia 41522, Egypt*
.

*Corresponding author: email aa.elzwawy@nrc.sci.eg; elzwawy1@gmail.com

Table 1S. *The assessed crystallinity of the prepared nanomaterials*

nanomaterials	*$Area_C$ (crystalline peaks)*	*Area (all peaks)*	*Crystallinity (X_C) %*
**CNTs**	1845.24	5187.4	26.23
**$Li_2Fe_3O_5$ (LFO)**	2093.6	5152.6	28.89
**CNTs/LFO (0.5%)**	2592.4	6056.7	29.97
**CNTs/LFO (1%)**	3117.5	6451.5	32.57
**CNTs/LFO (2%)**	2746.9	5913	31.71

Table 2S. *The structural parameters computed for the nanoparticles that were synthesized*

structure	D_v(nm) From Sherrer's analysis	δ (nm^{-2}) dislocation density	D_v(nm) From W-H analysis	(ε) Strain from W–H analysis
**CNTs**	2.5	0.16	2.1	1
**$Li_2Fe_3O_5$ (LFO)**	23.73	0.0017	22.5	0.06882
**CNTs/LFO (0.5%)**	24.42	0.00167	23.4	0.53
**CNTs/LFO (1%)**	24.35	0.00168	23.5	0.51
**CNTs/LFO (2%)**	24.49	0.00166	23.9	0.03713

Table 3S. *The magnetic parameters received from the magnetic hysteresis loop*

Structure	Saturation magnetization (emu/g)	Hc (left) (G)	Hc (right) (G)	Coercivity (G)	Remanent magnetization (emu/g)	Squareness
**CNTs**	0.6	-691.3	1109.2	900	0.187	0.311
**LFO**	35.77	-16.30	27.35	21.83	3.18	0.088
**CNTs/LFO (0.5%)**	14.40	-21.64	40.58	31.11	1.35	0.093
**CNTs/LFO (1%)**	20.95	-35.21	33.73	34.47	4.5	0.214
**CNTs/LFO (2%)**	25.07	-17.14	78.64	47.89	2.29	0.091

Table 4S. *The modified electrodes using the produced nanomaterials yielded the electrochemical parameters (CV & EIS). These values are taken from the impedimetric and voltammetric studies mentioned above.*

**Electrode type**	I_a (µA)	I_c (µA)	$E_{oxd.}$ (V)	$E_{red.}$ (V)	ΔE_p (V)	R_s (Ω)	$R_{ct (1)}$ (Ω)	**C (µF)**	**W (Ω)**	**SC (F/g)**
**CNTs**	225.6	-238.6	0.313	0.0015	0.157	74.6	80.5	121.6	171.4	121.61802
**LFO**	127.04	-135.12	0.334	0.0152	0.174	33.4	1150.3	72.5	189.6	52.54385
**CNTs-LFO 0.5**	349.5	-357.6	0.329	-0.040	0.144	112.6	50.3	136.2	72.6	118.02871
**CNT-LFO 1.0**	179.01	-192.10	0.22	0.0711	0.144	214.3	205.9	112.5	94.2	88.89111
**CNTs-LFO 2.0**	168.6	-176.7	0.356	-0.102	0.127	391.8	700.9	95.3	168.2	83.83291

Table 5S*. Comparison for different electrodes used for hydrogen peroxide (H_2O_2) detection, based on applied potential, linear range, and detection limit.*

**Materials**	**Electrode type**	**Buffer**	**pH**	**Applied Potential**	**Linear range (µM)**	**Detection Limit (µM)**	**Ref.**
**CNT-$NiCo_2O_4$**	SPE	PBS	7.4	0.7	2.5-275	0.01	[1]
**$NiCo_2O_4$/RGO**	GCE	PBS	7.0	-0.4	5-3000	0.41	[2]
**$MnCo_2O_3$/CNTs**	SPE	PBS	7.4	0.7	0.1-180	0.1	[3]
**rGo-Pt**	GCE	PBS	7.0	-0.08	0.5-3475	0.2	[4]
**Co_3O_4**	SPE	-	-	1.0	0.1-50	0.145	[5]
**$CoFe_2O_4$/ CNTs**	GCE	PBS	7.0	0.3	0.5-50	0.05	[6]
**Pt/rGO-CNT**	Paper electrode	-	-	-0.25	0.1-25	0.1	[7]
**Pol(azureA)-PtNPs**	SPE	PBS	7.4	0.1	0-300	0.052	[8]
**Co_3O_4-rGO**	GCE	-	-	-0.19	15-675	2.4	[9]
**CNTs/LFO**	SPE	PBS	7.4	0.7	0.01 -500	0.005	This work

Table 6S. Peroxide detection in real waste water samples using CNTs/LFO sensor.

peroxide concentration µM		RSD (%)	Recovery (%)
Added	Found		
5	5.1	0.19	102
10	9.8	0.62	98
20	19.9	1.38	99.5
30	29.7	2.96	99

References :

1 El-shazly M. Duraia a, B. M. Adebiyi, S. Das, H. S. Magar, G. W. Beall and R. Y. A. Hassan, *Phys. E Low-dimensional Syst. Nanostructures*, 2024, **159**, 115902.

2 M. Wang, C. Wang, Y. Liu, B. Hu, L. He, Y. Ma, Z. Zhang, B. Cui and M. Du, *Microchim. Acta*, 2020, **187**, 436–450.

3 Nehad Yousf, Emtinan Ouda, Hend S. Magar, Rabeay Y. A. Hassan, S.A. Mansour, El-Shazly M. Duraia, *J. Electrochem. Soc.*, 2022, **169**, 47518.

4 Y. Zhang, X. Bai, X. Wang, K. K. Shiu, Y. Zhu and H. Jiang, *Anal. Chem.*, 2014, **86**, 9459–9465.

5 S. Barkaoui, M. Haddaoui, H. Dhaouadi, N. Raouafi and F. Touati, *J. Solid State Chem.*, 2015, **228**, 226–231.

6 S. Sahoo, P. K. Sahoo, S. Manna and A. K. Satpati, *J. Electroanal. Chem.*, 2020, **876**, 114504.

7 Y. Sun, K. He, Z. Zhang, A. Zhou and H. Duan, *Biosens. Bioelectron.*, 2015, **68**, 358–364.

8 R. Jiménez-Pérez, J. González-Rodríguez, M. I. González-Sánchez, B. Gómez-Monedero and E. Valero, *Sensors Actuators, B Chem.*, 2019, **298**, 126878.

9 L. Kong, Z. Ren, N. Zheng, S. Du, J. Wu, J. Tang and H. Fu, *Nano Res.*, 2015, **8**, 469–480.
